# Crystal field splitting and spontaneous polarization in InP crystal phase quantum dots

**DOI:** 10.1038/s41598-022-19076-w

**Published:** 2022-09-16

**Authors:** Martyna Patera, Michał Zieliński

**Affiliations:** Institute of Physics, Faculty of Physics, Astronomy and Informatics, Nicolaus Copernicus University in Toruń, Toruń, Poland

**Keywords:** Nanoscale devices, Materials science, Condensed-matter physics, Theory and computation

## Abstract

Crystal phase quantum dots are formed by vertically stacking zinc-blende and wurtzite phases during nanowire growth. In this work, we show, using an atomistic many-body approach, that crystal field splitting in the wurtzite phase, as well as spontaneous polarization originating from the phase interfaces, will strongly affect the properties of lowest hole states in InP crystal phase quantum dots, and in turn the excitonic optical spectra. We also show that the artifact-free modeling of crystal phase quantum dots should incorporate any additional potentials on equal footing with the electron-hole interaction. In this paper, we discuss a reliable theoretical framework that can be applied to investigate the electronic and optical properties of InP-based crystal phase quantum dots. The importance of accurate excitonic calculations for such systems is highlighted in view of their potential applications in nanowire photonics, yet further research is necessary for bringing theory and experiment in agreement.

## Introduction

**Figure 1:**
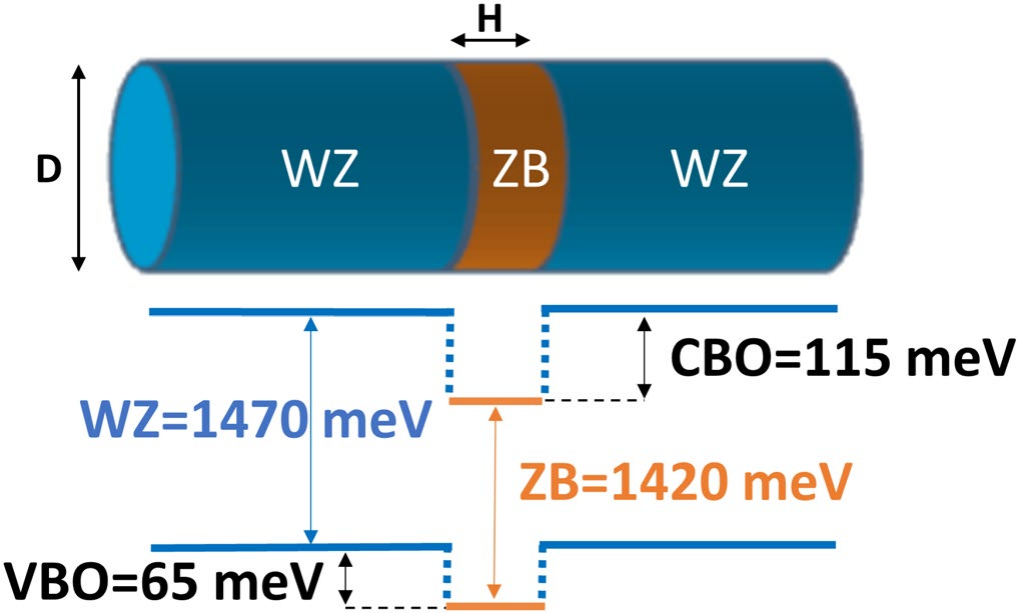
Schematics of InP crystal phase quantum dot under consideration, formed by zinc-blende (ZB) section embedded in wurtzite (WZ) nanowire, including band alignment of InP crystal phases. H and D denote CPQD height and diameter, respectively.

A single crystal phase quantum dot (CPQD)^1^ is a nanostructure with type-II confinement, where electrons can be confined in the zinc-blende (ZB) section of the nanowire, whereas holes occupy the host wurtzite (WZ) nanowire^2^. Crystal phase-defined systems or polytype homostructures^3^ (a.k.a. heterostructures^4^) have atomically sharp interfaces with no intermixing, making them ideal candidates for a broad range of applications, e.g., as single-photon sources with very strong photoluminescence signals^5^ and photon indistinguishably^6^. The stacking faults (i.e., ZB sections in WZ nanowires or vice versa) could be considered detrimental for typical nanowire quantum dots by reducing their high spectral purity^7^. Contrarily, CPQDs exploit the fact that crystallographic structures do not intermix within a monolayer^8^, allowing for geometry control with the precision of a single atomic layer^9,10^. Moreover, a switch between a cubic (ZB) and hexagonal (WZ) lattice defines a confining potential (Fig. 1) because the crystal phases have different band structures^1,2,6,11–16^. All these allow InP crystal phase quantum dots to be a source of pure single-photons and cascaded photon-pairs with excellent optical properties^10^, and can open new possible routes to advanced entangled photons generation^9^.

Here, using atomistic tight-binding self-consistent calculations, augmented with configuration interaction treatment of correlation effects, we demonstrate that the crystal field splitting in the WZ phase^13,14^ (due to the breaking of the degeneracy of states in a hexagonal lattice) and the spontaneous polarization^9,17–19^ (due to the relative displacement between the cations and anions in a non-ideal hexagonal lattice) can both strongly affect the structure of hole states in CPQDs, and in result strongly affect their excitonic optical spectra. Although relatively weak, the crystal field splitting will impact the multi-band character of hole states by shifting light-hole states deep into the hole spectra and leaving the ground hole state of heavy-hole character, with an in-plane polarization of the emitted light. On the other hand, we found that the spontaneous polarization can be considered, as a potential mechanism causing the “mixing” of delocalized hole states, thus leading to peculiar double-peak features in the excitonic spectra for CPQDs^10^.

## Results

Figure 1 shows schematics of a CPQD formed by a section of ZB embedded in a host WZ nanowire. The ZB fragment forms a well for the electron state, and it forms a barrier for hole states leading to a type-II confinement situation.

**Figure 2:**
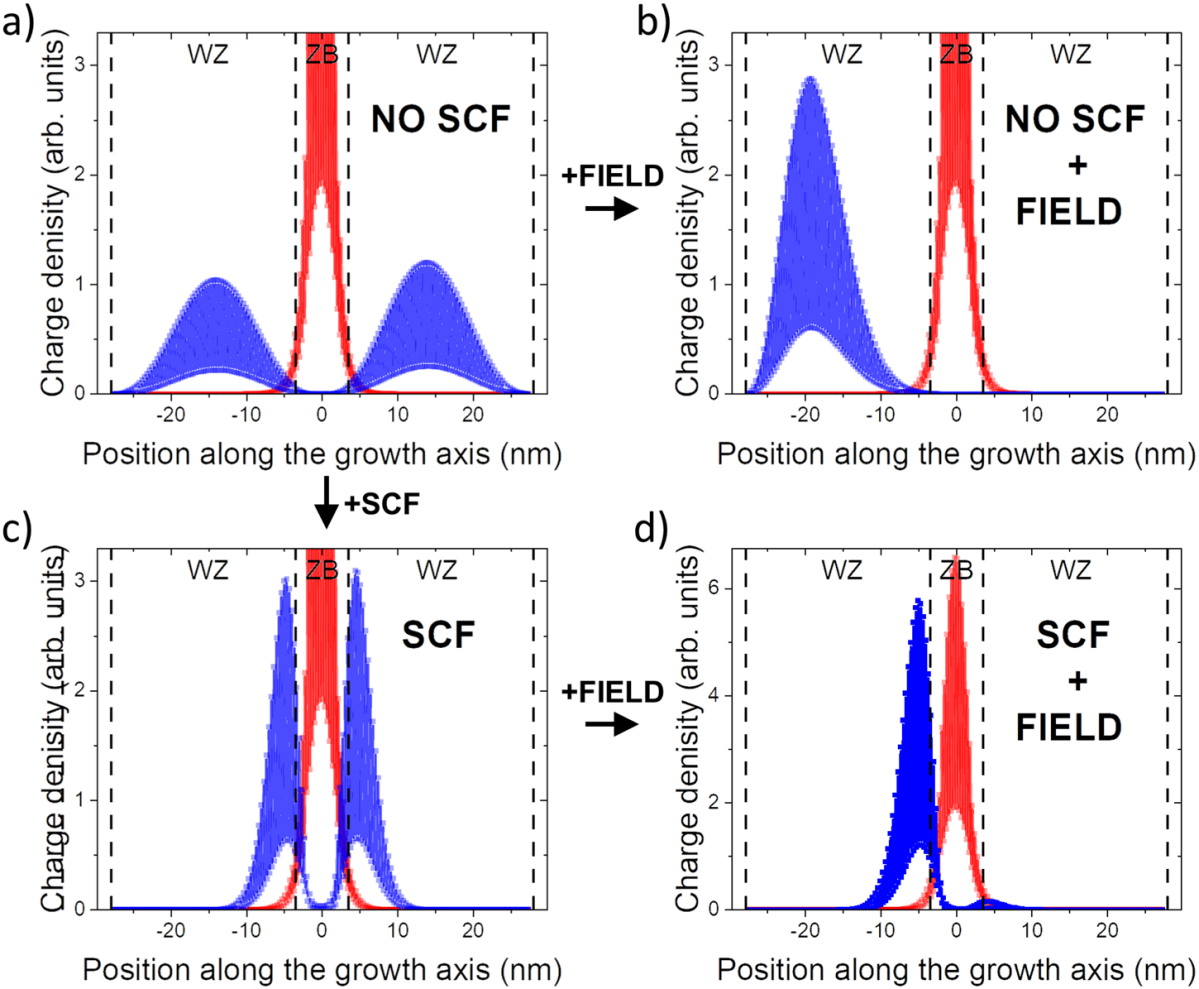
The charge density of ground hole (blue) and ground electron (red) states calculated for a CPQD of H = 4 nm and D = 23.5 nm, for several different cases: (**a**) with no electron-hole interaction, (**b**) with no electron-hole interaction and an external electric field forming a well for holes at the edge of the computational box, (**c**) with electron-hole interaction accounted for via self-consistent approach discussed in the text, (**d**) with electron-hole interaction and external-field taken on equal footing. The electron state remains virtually identical in all cases. Note different vertical scales (**a**–**c**) that truncate the top of the electron peak for better hole state visualization. Vertical dashed lines mark the edges of WZ sections.

This is illustrated in Fig. 2, where the ground hole state charge density is presented for a CPQD with H = 4 nm (4 single ABC stacking sequences), with a diameter of D = 23.5 nm (40 lattice InP constants), which (unless specified otherwise) is studied throughout this paper. Interestingly, hole states delocalize over the available WZ nanowire volume, Fig. 2 (a) (top-left corner), and only weakly penetrate into the ZB section where the ground electron state resides. To be able to calculate the excitonic properties of such a system, in a typical semi-empirical approach^20^, one would first calculate several lowest single-particle electron and hole states, corresponding to a non-interacting case, and only then run excitonic calculation by solving a many-body Hamiltonian expanded in a basis of these states (exact diagonalization or configuration interaction (CI) approach). Such an approach is still possible for CPQDs;^2,10,21^, however, it may be challenging since hole states strongly depend on the size of the assumed computational box (or the nanowire section lengths), and one may face troubles with CI convergence. These problems could be even more severe with an increasing size box, an increase of which is typically a way of dealing with computational domain convergence problems^22^. Further problems could occur when one would like to incorporate the effect of an external electric field (Fig. 2b) or an additional (e.g., due to spontaneous or piezoelectric polarization) potential that might be present in the system.

In the case of electric-field, without electron-hole interaction included, there is no confinement for a hole other than due to the computational box. Thus with the applied field, the hole state will “leak out“ (Fig. 2b) to the edges of a computational domain where an “artificial“ quantum well is formed. This problem will occur irrespectively of sign and magnitude of applied bias. It will be even further magnified for larger computational boxes (longer section of WZ nanowire included in the simulation), which otherwise would be a solution to domain size problems^22^. Such hole states, as shown in Fig. 2b, would form an inferior basis for CI calculations, with no chances of convergence in a practical calculation, where the size of the single-particle basis is typically strongly limited.

As a solution to that problem, we account for electron-hole interaction already at the step of tight-binding calculation (see Methods), resulting in hole states experiencing the presence of confining Coulomb potential due to an electron (Fig. 2c), which leads to charge redistribution, and strongly affects the spatial localization of the hole, as compared to a non-interacting case. Such inclusion of electron-hole interaction preceding the CI computations will not only significantly improve the overall description of electron-hole interaction as discussed later but will also allow for spurious-states-free treatment of external fields or additional potentials (Fig. 2d).

**Figure 3:**
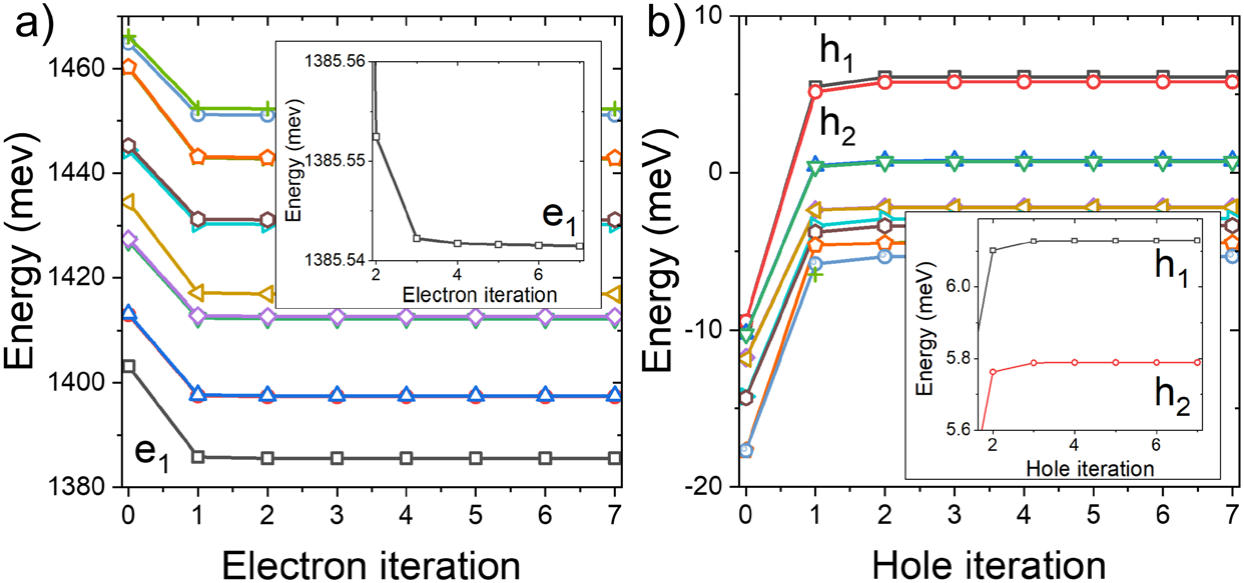
Several lowest electron (**a**) and hole (**b**) states energies, for a CPQD of H = 4 nm and D = 23.5 nm, during the self-consistent treatment of electron-hole interaction. The rapid initial step is due to the inclusion of interaction (as compared to the non-interacting zeroth iteration). Then the process converges rapidly. The inset shows the magnification of the evolution for the ground electron level ($e_1$) and the first two lowest hole states ($h_1$/$h_2$).

The incorporation of electron-hole interaction at the tight-binding (TB) stage comes with a penalty related to the necessity of running several tight-binding calculations to achieve convergence in a self-consistent process. This is shown in Fig. 3, where energies of several lowest electron and hole states are given as a function of the number of iterations (electron and hole calculations). Thus compared to a typical TB-CI scheme, where one performs only a single TB calculation for a given system, in an SCF-CI procedure, one performs several additional TB computations, which are thus more computationally challenging. Nonetheless, we found that only several iterations (typically a total of 10 tight-binding calculations) will lead to electron and hole energies converging well within the sub-meV error bar. We also note that due to the use of efficient solvers^23^, the entire process scales linearly with a number of atoms, and modern parallel computers (with hundreds of CPU cores) allow to perform a single TB calculation for a 1 million atom system within an hour.

**Figure 4:**
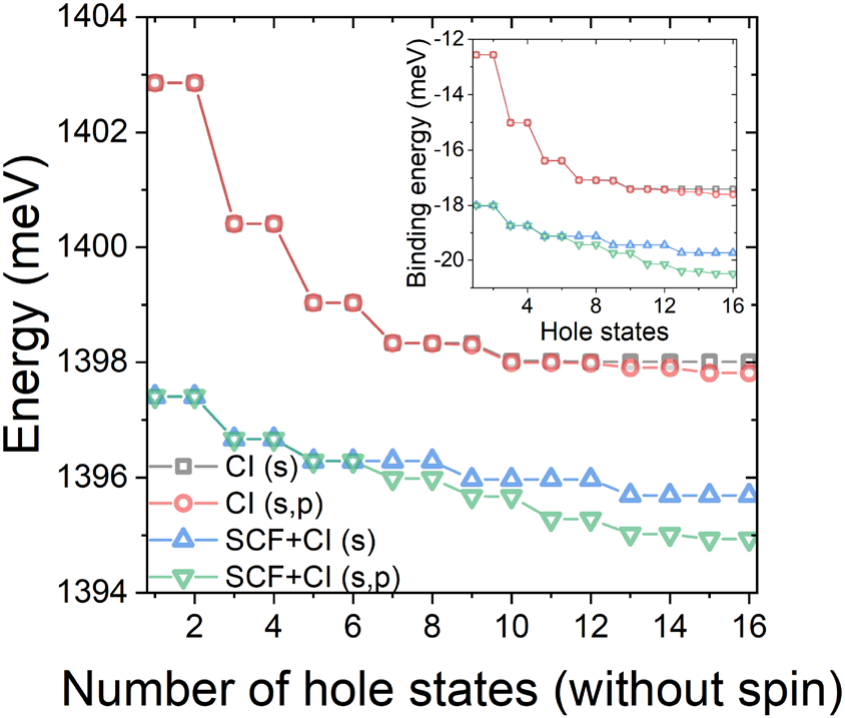
The ground exciton state for a CPQD of H = 4 nm and D = 23.5 nm, calculated in different approximations and as a function of hole states included in the CI modeling. The inset shows the exciton binding energy. (s) and (p) refer to a number of electron states, 1 and 3 respectively (2 and 6 with spin), included in the CI calculation. Each hole state is actually a pair of spin (Kramers) degenerate states.

Whereas potentially time-consuming, these additional computational efforts are not only necessary for the incorporation of additional fields (where a plain TB-CI approach would diverge) but also bring benefits to a case with no external field. This is shown in Fig. 4, where the ground exciton state energy, and the binding energy (calculated with respect to single-particle $e_1-h_1$ gap), are presented as a function of hole states accounted for in the CI calculation (see the Methods) that was run on top of a case with and without the SCF treatment of electron-hole interaction. As shown in Fig. 4, for a ground excitonic state calculation, it may be actually more beneficial to run SCF electron-hole calculation even without the CI (i.e., number of hole states equal to 1) than to neglect the self-consistency at all and simply increase the number of CI configurations (by including higher hole states). Namely, the corresponding binding energy is equal to − 18 meV for a single electron-hole configuration with SCF, thus comparable to − 17.6 meV obtained with no SCF and as many as 192 electron-hole configurations (6 electron times 32 hole states with spin). Nonetheless, adding CI for SCF states is still worth accounting for, as it further reduces the binding energy to − 20.5 meV. We also note the evolution of excitonic energies has a step-like character, which reflects the character of hole states, which come in pairs of quasi-bonding/antibonding nearly degenerate states (e.g., $h_1$/$h_2$) with minor, sub-meV splitting. We further note here the inclusion of higher electron states (two states of *p* character, as compared to the ground electron *s* state) is meaningful only if a sufficient number of hole states is included. Finally, we comment that the inclusion of an even larger number of electron and hole states in the CI calculation is prohibited here by the increasing complexity of the electron-hole Coulomb matrix elements calculations^23^. We note that a substantial part of this complexity is related to the necessity of resolving higher-excited hole states which are energetically very closely spaced (quasi-degenerated), which is often a formidable challenge for sparse-matrix iterative solvers^24^.

### Crystal field splitting

The crystal field splitting in WZ is related to a relatively small splitting of A and B bulk hole bands due to underlying crystal lattice geometry^4,11,13,25^. However, the reported values of WZ crystal-field splitting (CFS) range from 26 to even 303 meV^2,11,13,26,27^.

**Figure 5:**
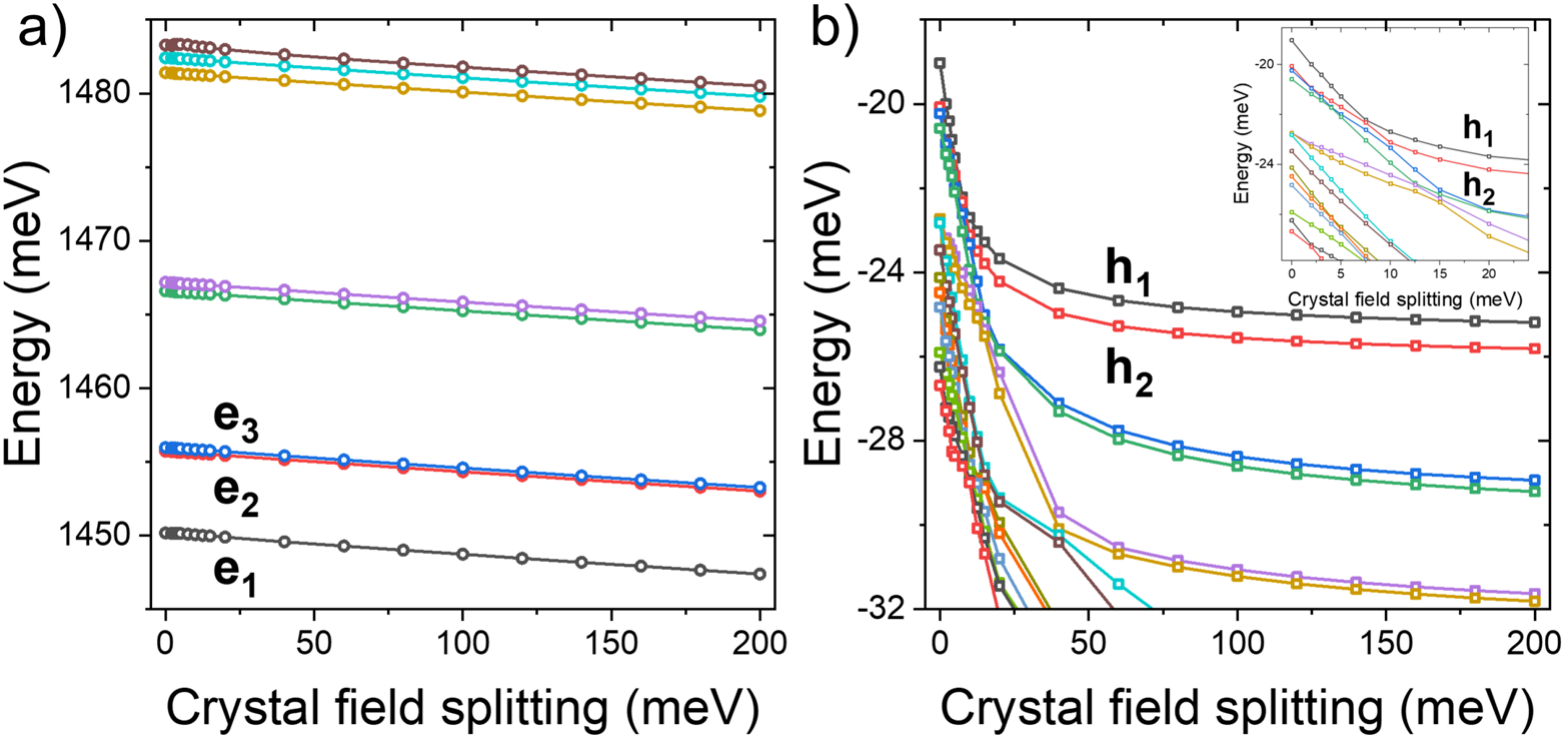
The effect of WZ crystal field splitting on the spectra of electron (**a**) and holes (**b**). Whereas the electron shows a relatively negligible drift under the inclusion of WZ crystal field splitting, the detailed structure of the hole state changes dramatically, especially if compared to a case with a crystal field neglected (see the inset).

As the crystal field splitting is not reliably known for the WZ InP phase, it is tempting to neglect it entirely to avoid ambiguity^10^. However, as shown in Fig. 5, for a CPQD with H = 1 nm and D = 23.5 nm, the presence of the crystal field has a very strong impact on the level structure of hole states. Here, we calculated the energy structure of electron and hole states by artificially changing the value of WZ crystal field splitting. As electron states are predominately localized in the ZB section, the WZ crystal field rather weakly affects their spectra. However, the electron has non-negligible charge density (wave function tails as shown earlier in Fig. 2) in the WZ region, and in the multi-band TB treatment conduction band state is by definition not decoupled from the valence band, so thus a change in hole bulk-band structure may, in turn, affect the electron state. Nonetheless, the only apparent effect of the crystal field on the electron is related to a relatively weak red-shift of electron energies as the crystal field increases. However, as noticed earlier, the situation is very different for hole states, especially for a range of crystal field splitting values below approximately 20 meV (the inset on Fig. 5), where the spectra change substantially, with several hole states (including the hole ground state) that are profoundly red-shifted by the presence of the crystal field.

**Figure 6:**
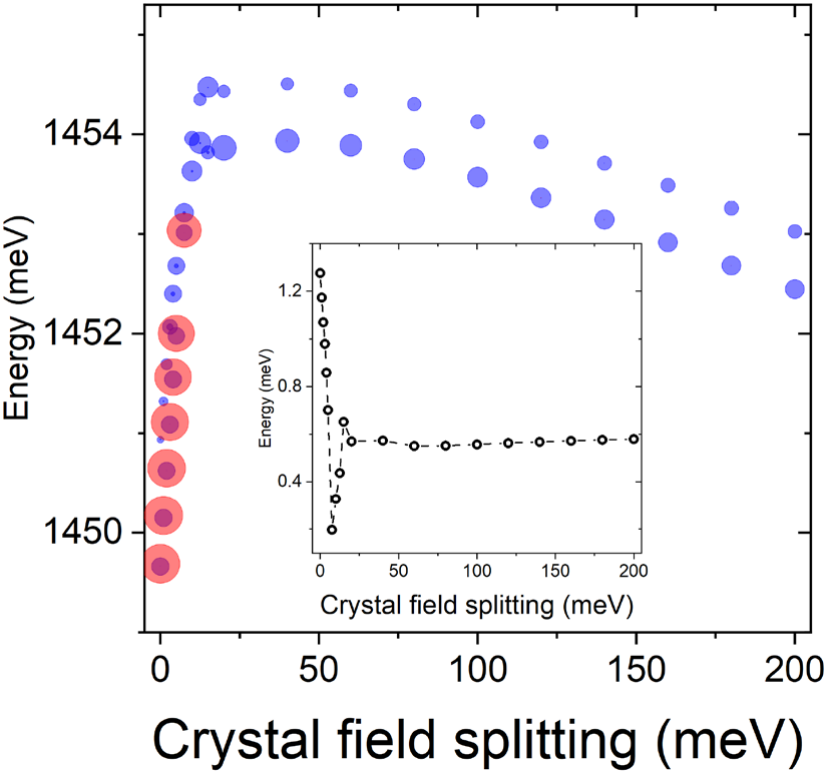
Energy spectra of the lowest two excitonic branches for a crystal phase quantum with H = 1 nm, D = 23.5 nm. The size of each circle corresponds to the state’s optical activity. The red color for small crystal field splitting values corresponds to out-of-plane (z) polarized (along the nanowire) emission, whereas the blue color corresponds to the in-plane (x/y) polarization of emitted light. The inset shows the energy spacing between lower and higher exciton branches (manifolds).

Figure 6 shows the corresponding excitonic spectra calculated in a basis of 1 (2 with spin) electron and 4 (8 with spin) hole states studied earlier in Fig. 5. As the structure of the hole state changes substantially with the application of the crystal field splitting, so could, in principle, the excitonic energy. However, the energy difference between a case with artificially neglected crystal field splitting (i.e., 0 meV) and crystal field splitting of 150 meV is on the order of 2 meV, thus relatively small as compared to other factors, e.g., the uncertainty of WZ band gap^13,26,28^ or CI convergence problems mentioned earlier. Yet, the optical spectra of the exciton, with and without the inclusion of crystal field splitting, are very different. Without the crystal field splitting, the ground hole state is apparently of a light-hole character leading to z-polarized (along [111] ZB/[0001] WZ direction) emission from the nanowire (as heavy-hole states can’t have z-polarized emission due to selection rules). At the intermediate range of crystal field splitting values, the structure of hole states and their excitonic spectra are quite unstable (see the inset) due to numerous level crossings in the hole spectra. Finally, the spectra stabilize for splitting larger than 40 meV, and the ground hole/excitonic state is of heavy-hole character with in-plane polarized emission. The excitonic spectra have a characteristic structure of two peaks (a pair of blue circles in Fig. 6) corresponding to dominant emission from the lowest excitonic branch (a manifold of four states—two dark and two bright—due to electron-hole exchange interaction), which is constituted predominately by electron-hole configurations involving $e_1$ and $h_1$ states (i.e., four states with spin). Somewhat weaker emission is also present in optical spectra of the higher excitonic branch (formed by four states constituted predominately by configuration involving $e_1$ and $h_2$). Both lower and higher branches are in-plane polarized, with no fine structure splitting due to $C_{3v}$ symmetry. However, the final polarization of light measured experimentally may strongly depend on the polarization proprieties of a host nanowire^29,30^. Such double peak features in the excitonic spectra are very characteristic for CPQDs^10^, yet their origins could be attributed to many factors lowering system symmetry. We note, however, that for the case considered here, due to the significant energy separation between two excitonic branches, in a typical, low-temperature photoluminescence experiment, the higher branch would not be optically observable due to weak thermal occupation probability^31^. Finally, we emphasize that although the results presented in Figs. 5 and 6 were obtained for a particular quantum dot system, the conclusion regarding the role of spontaneous polarization is general in CPQDs, as we found very similar behavior and findings, for quantum dots of different heights and diameters, with and without self-consistency accounted for, as well as for other CI basis, etc. To summarize, the inclusion of the crystal field is essential for understanding the optical activity of exciton in CPQDs and must be included in the modeling; however, the choice of the actual parameter does not play a crucial role as long as it is chosen within reasonable intermediate values reported in the literature^4,11,25^.

### Spontaneous polarization

The spontaneous polarization occurs naturally in WZ materials, and it particularly plays a fundamental role in nitrates^11,32,33^. Although much smaller in III–V materials, it could strongly affect GaAs and GaP polytype systems; however, its role is not fully understood^9,14,18,34^. For InP WZ, the situation is further complicated, as there are very few reports of $P_{SP}$ spontaneous polarization magnitude^12,19,35^, with a pronounced uncertainty regarding the magnitude and sign^14^, yet with an absolute value of $P_{SP}$ likely not exceeding $10^{-3}$ C/m$^2$. Thus, here we decided to study the potential impact of spontaneous polarization by varying it from − 0.5 to 0.5 $\times 10^{-3}$C/m$^2$.

**Figure 7:**
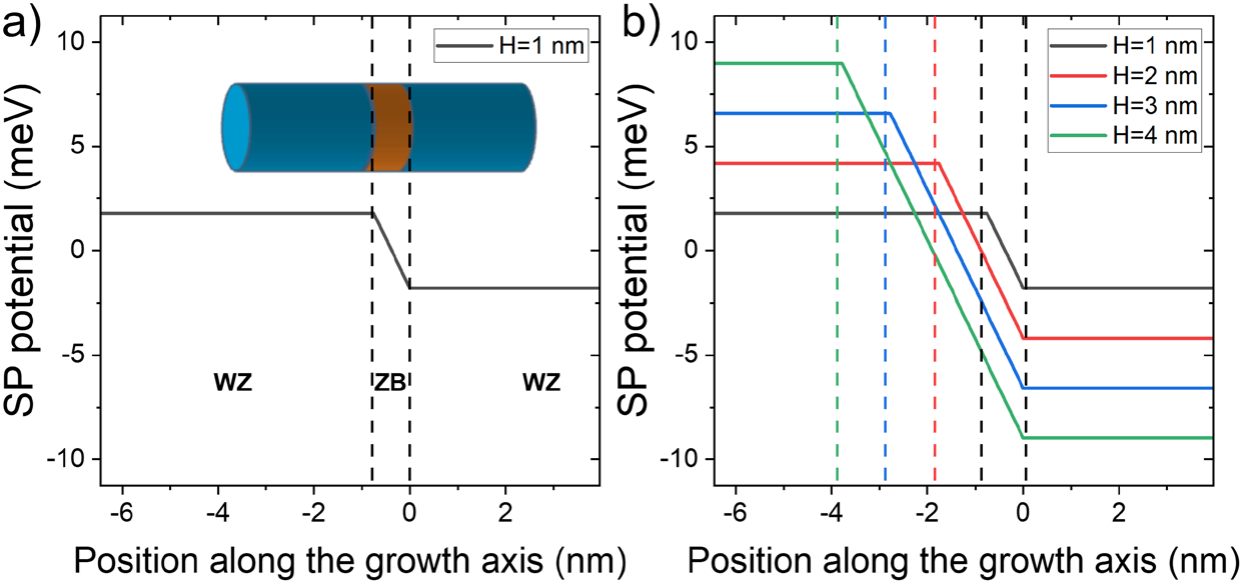
(**a**) spontaneous polarization (for $P_{sp}=0.5 \times 10^{-3}~$C/m$^2$) potential profile along nanowire [111] growth axis. The spontaneous polarization leads to a step-like potential with a triangular-like quantum well in the ZB region and potential bias between both WZ sections. (**b**) the potential step increases (as spontaneous polarization charge builds up) with the increasing quantum dot height (ZB region thickness).

Despite being relatively weak, even such small spontaneous polarization may impact the spectra of CPQDs. This is due to the shape of the spontaneous polarization potential, as shown in Fig. 7, which leads to a pronounced bias between WZ sections and field gradient in the ZB region. We note that proper (spurious states free) incorporation of this field into modeling is possible only if the electron-hole interaction and the field are treated on equal footing, as described earlier.

**Figure 8:**
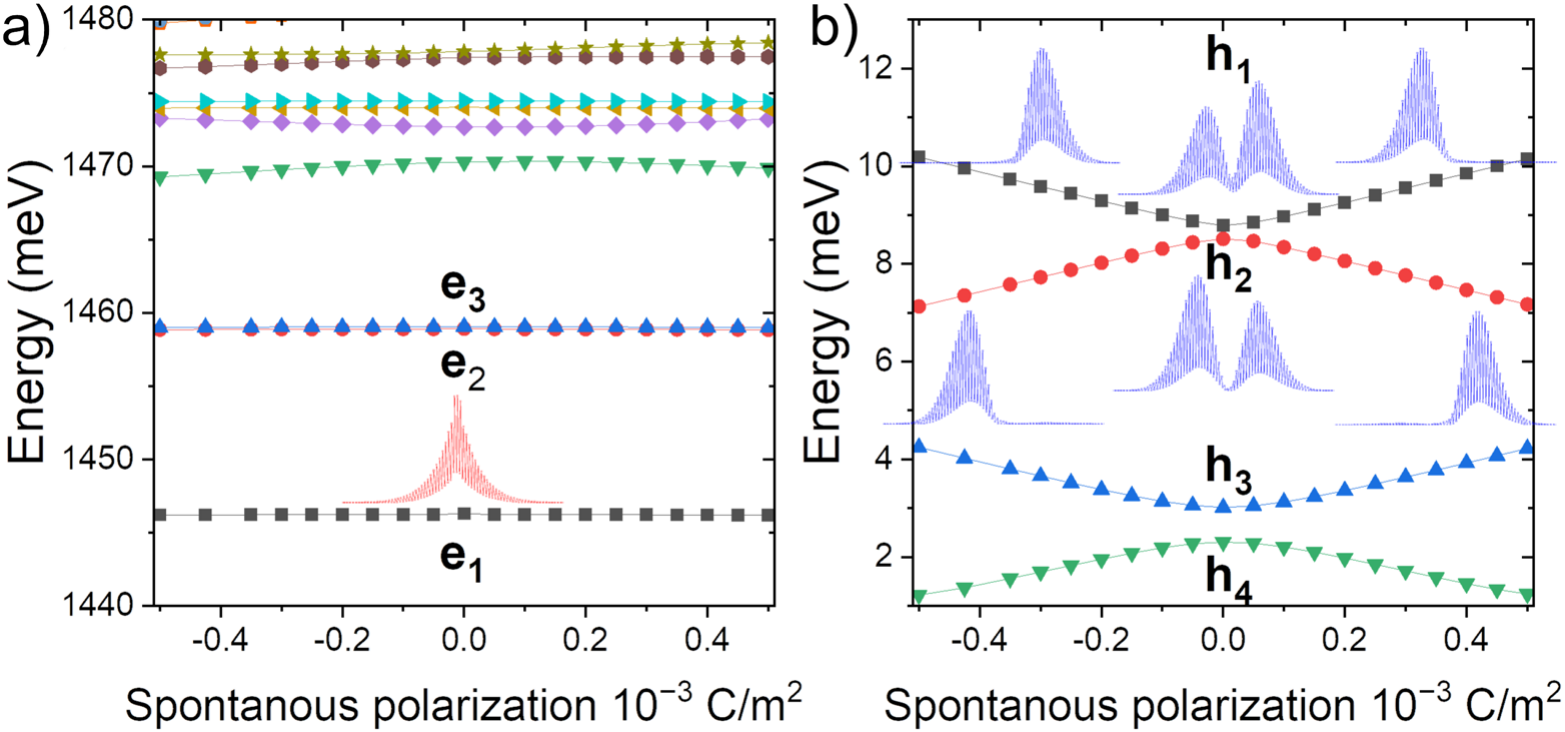
The evolution of (**a**) electron and (**b**) hole levels as a function of spontaneous polarization. The electron energy spectra remain rather immune to the polarization, where the field strongly splits hole states. The miniatures show corresponding charge densities for the electron ground state ($e_1$), which virtually is not affected by the spontaneous polarization, and for $h_1$/$h_2$ states which are strongly spatially separated by the field.

The spontaneous polarization potential thus leads to the formation of a triangular-like well in the ZB section. Therefore one expects (in the framework of first-order perturbation theory) that (for small fields) the energy correction due to spontaneous polarization to electrons states (of even parity along [111] ZB direction) would nearly vanish. Its overall impact on electron states should thus be relatively small. This expectation is justified by the results of calculations as shown in Fig. 8, where both the ground electron states $e_1$ and the first two excited electron states $e_2$/$e_3$ are practically immune to the presence of an electric field due to spontaneous polarization. Contrarily, the hole states are strongly affected by spontaneous polarization. Even for the smallest considered $P_{SP}$, there seems to be a pronounced effect on the $h_1$/$h_2$ levels splitting. This is again expected since hole levels spacings are generally much smaller in CPQDs than electron spacings, and spontaneous polarization introduces a strong, effective bias between “left”/“right” (or “up”/“down”) WZ parts of the InP nanowire.

**Figure 9:**
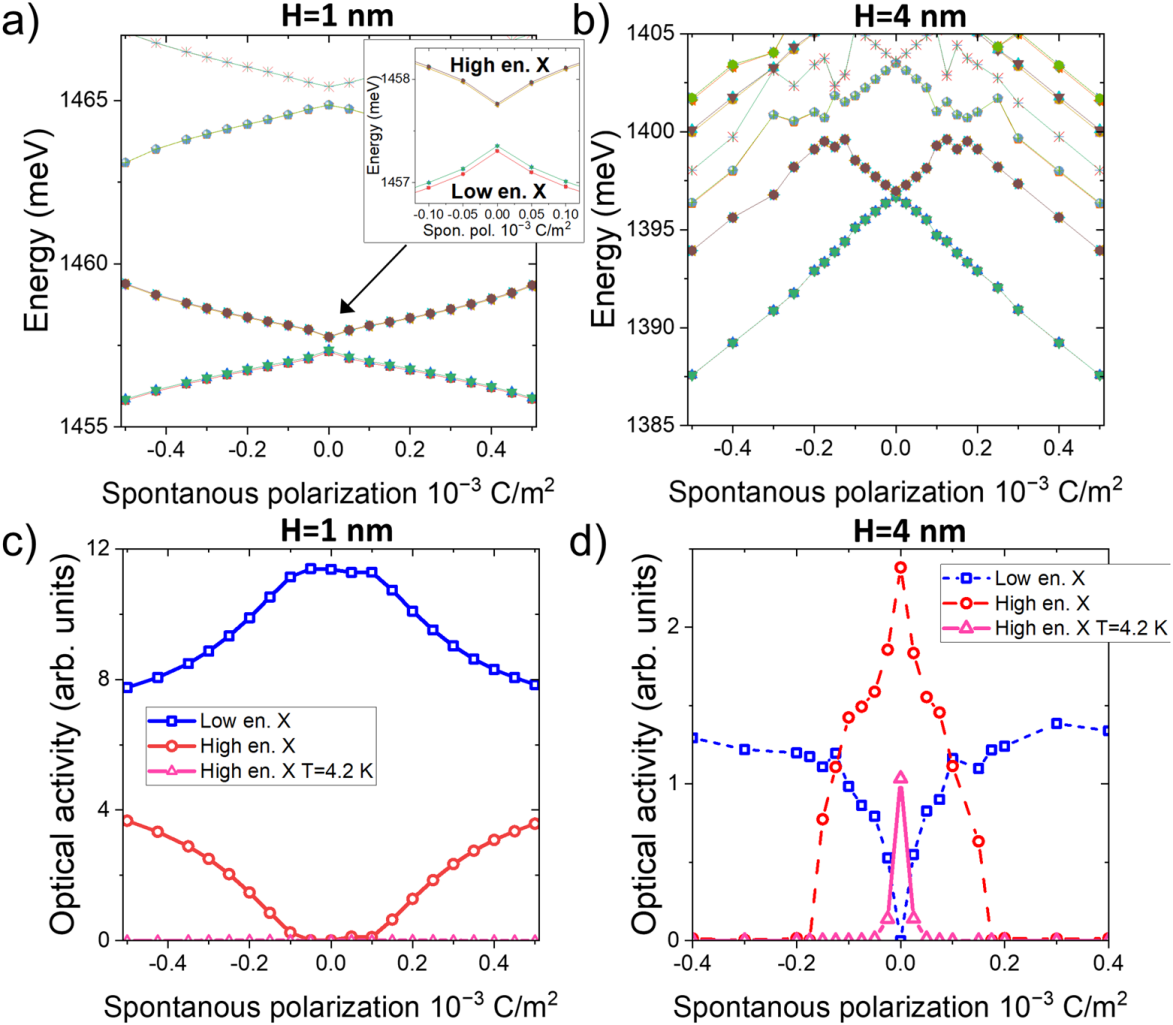
The energy spectra (upper row) and optical activity (lower row) of several lowest excitonic levels for CPQD of different heights (**a**,** c**) H = 1 nm and (**b**,** d**) H = 4 nm, and the same diameter D = 23.5 nm, as a function of spontaneous polarization. Magenta (triangles) describe—strongly quenched—optical activity of higher excitonic branch when low occupation due to low experimental temperature (4.2 K) is accounted for. Note that the dominant optical activity of a higher excitonic branch for a larger dot (H = 4 nm) is due to the antibonding ground hole state for a case with no spontaneous polarization. The inset magnifies low and high excitonic branches with (weakly) visible exchange splitting within the branches (the lowest two states in each branch are always dark to spin-selection rules).

As the spontaneous polarization affects the hole states, it should impact excitonic states, as shown in Fig. 9, where it first leads to increased energy separation of the two lowest excitonic branches (Fig. 9a). This effect is even more pronounced for a larger dot, where the built-up of spontaneous polarization potential is even larger, with the onset of the higher-excited hole states that rapidly drop their energies with the increasing polarization. This is related to the underlying evolution of the hole state and effective (due to field) separation of the system into two halves. For a sufficiently large spontaneous polarization, the lowest hole state will “migrate” into the area which is more energetically favorable (left or right depending on $P_{SP}$ sign).

We note that the results remain practically symmetrical with respect to a sign. However, there is a tiny asymmetry related to a lack of system inversion (in the growth direction) due to [111] ZB/[0001] WZ growth. We also note that the complicated energy behavior of higher excitonic states (oscillatory patterns) is related to level crossings of excited hole states and is also due to issues of CI convergence with respect to these states.

Finally, Fig. 9c and d show the corresponding optical activity of excitonic states. For “flat“ H = 1 nm CPQD, the ground hole $h_1$ state of a bonding-like character, namely, could be very well described by a simplistic picture of a symmetric, linear combination of left and right WZ states. Thus the lower excitonic manifold (we reiterate that these are four states, split by the exchange interaction), formed predominately from configurations involving $e_1$ (two states with spin) and $h_1$ (two states with spin), is optically active. Contrarily, the first excited hole state resembles very much an antibonding (anti-symmetric) combination of hole states from two WZ sections, and thus it remains optically inactive. We also note that the notion and analysis of bonding/antibonding hole states in InP CPQDs have been thoroughly studied in our recent work published elsewhere^36^. For the H = 4 nm system, the ordering of hole states is reversed, with the ground hole state $h_1$ having however peculiar antibonding character^37–40^ and thus odd parity preventing optical transitions for the lowest excitonic branch dominated by $h_1$. On the other hand, the higher excitonic branch is optically active as $e_1$-$h_2$ recombination is optically allowed (due to $h_2$ bonding character and even parity).

In both cases (H = 1 nm and H = 4 nm), the spontaneous polarization will effectively “mix“ quasi-molecular^10^ hole states, thus shifting their character from bonding-antibonding states occupying both WZ sections surrounding the ZB part, to hole states localized on either side of the ZB section. Thus, going from pure bonding-antibonding states to left/right localized holes could be viewed as mixing quasi-molecular states due to a spontaneous polarization field. In effect, the higher excitonic branch will gain some oscillator strength for H = 1 nm, whereas the higher excitonic branch will lose some strength for H = 4 nm. Thus the presence of the spontaneous polarization, as any other factor in mixing holes states such a strain^36^, could lead to the onset of double peak features in the excitonic spectra. Again we emphasize that in a relevant photoluminescence experiment, the optical activity of excitons would be affected by optical dipole moments and temperature, which determines the populations of excitonic states^31^. For a 4.2 K experimental scenario and the H = 1 nm system shown in Fig. 9c, the higher branch would not be occupied due to the significant energy separation from the excitonic ground state. Contrarily, for H = 4 nm (Fig. 9d) such a double-peak feature would appear for a very small spontaneous polarization. However, it would be rapidly quenched for larger (though still relatively small) $P_{SP}$ values. The further increase of spontaneous polarization field, above $10^{-3}$ C/m$^2$, would lead to an increased spatial electron-hole separation and a further decrease of InP CPQD activity. However, available experimental results for InP systems do not suggest any such reduction.

## Discussion

In this work, we show that both the crystal field splitting in WZ and the spontaneous polarization originating from the WZ-ZB-WZ interface may play an essential role in the excitonic spectra of InP CPQDs. Although the neglect of the crystal field splitting in the theoretical modeling will only weakly affect excitonic energies, it will have a pronounced impact on the excitonic optical spectra, affecting the polarization of the emitted light in particular. We also show that theoretical modeling of CPQDs systems must likely involve accounting for electron-hole interaction already at the stage of quasi-particle calculations. Specifically, incorporating the Coulomb interaction when modeling hole states will lead to a substantial spatial redistribution of their probability charges. Neglecting to do so will not only affect a subsequent inclusion of correlation effects (via the configuration interaction approach) but may result in a lack of ability to properly incorporate additional potentials into the modeling, such as the spontaneous polarization or the external electric field. Moreover, although the spontaneous polarization is expected to be weak compared to, e.g., nitrate systems, we found it may strongly affect the hole spectra, break the spatial structure of bonding/antibonding (quasi-molecular) hole states, and, via that, affect the excitonic optical spectra of InP CPQDs as well. However, as we are modeling uncapped wires (as they are realized experimentally) and there are not sufficient experimental indications of spontaneous polarization in InP systems, there may likely exist a compensation mechanism of $P_{sp}$ charges (e.g., due to surface states) that need to be studied further^8,14,18^. Similarly, apart from the spontaneous polarization and crystal field splitting uncertainties, there is a significant ambiguity of several other vital bulk parameters for ZB-WZ mixed InP systems, including, e.g., the valence band offset and strain at the ZB-WZ interface^13,26,28^, as well as the piezoelectric potential that could result due to strain. These uncertainties will inevitably affect any empirical approaches for modeling InP polytype nanostructures, thus including, e.g., $k\cdot p$ methods and empirical pseudopotential frameworks as well. Thus, further ab-initio theoretical efforts and experimental research are very much needed to establish more accurately essential variables determining the properties of InP CPQDs. This step is necessary for correlating between structures assumed in theory with optoelectronic properties measured experimentally and for engineering future optoelectronic devices based on CPQDs.

## Methods

We model the quantum dots by a ZB InP segments of different heights, H = 1 and H = 4 nm (corresponding to one and four ABC stacking sequences) grown along [111] direction, sandwiched between two WZ InP segments along [0001]. The total length of the considered system is approximately equal to 60 nm, corresponding to 0.9 and 0.95 million atoms for H = 1 and H = 4 nm, respectively.

We use empirical tight-binding theory for the electron and hole states with an sp$^3$s$^*$ orbital model and nearest neighbors coupling. For the ZB InP section, we use Vogl et al.^41^ tight-binding parameters augmented to account for spin-orbit effects. The single-particle tight-binding Hamiltonian for a system of *N* atoms and *m* orbitals per atom can be written in the language of the second quantization as follows^20,42^:

1 $$\begin{aligned} \hat{H}_{TB} = \sum _{i=1}^N \sum _{\alpha =1}^{m} E_{i\alpha }c_{i\alpha }^+c_{i\alpha } + \sum _{i=1}^N \sum _{\alpha ,\beta =1}^{m} \lambda _{i\alpha ,\beta }c_{i\alpha }^+c_{i\beta } + \sum _{i=1}^N \sum _{j=1}^{near. neigh.} \sum _{\alpha ,\beta =1}^{m} t_{i\alpha ,j\beta }c_{i\alpha }^+c_{j\beta } \end{aligned}$$

where $c_{i\alpha }^+$ ($c_{i\alpha }$) is the creation (annihilation) operator of a carrier on the (spin-)orbital $\alpha$ localized on the site *i*, $E_{i\alpha }$ is the corresponding on-site (diagonal) energy, and $t_{i\alpha ,j\beta }$ describes the hopping (off-site and off-diagonal) of the particle between the orbitals on the four nearest neighboring sites. Index *i* iterates over all atoms, whereas *j* iterates over the four nearest neighbors only. Coupling to further neighbors is thus neglected, and $\lambda _{i\alpha ,\beta }$ (on site and off diagonal) accounts for the spin-orbit interaction following the description given by Chadi^43^, which includes the contributions from atomic *p* orbitals. For the WZ InP section, we have additionally modified the parameters from Vogl et al. ^41^ to account for the increased band gap and the valence band offset between the WZ and ZB segments^10^. We fit our tight-binding parameters to reproduce the wurtzite band gap (1.474 eV) and the WZ-ZB valence band offset (64.6 meV), as has been recently reported by De and Pryor^13^. The ZB band gap is set to 1.42 eV. The band alignment used in this work is thus shown in Fig. 1. The spin-orbit splitting is assumed to be identical in the ZB and WZ crystal phases and is equal to 126 meV. The crystal field splitting is accounted for by including an energy shift of (diagonal) onsite tight-binding matrix element in the WZ phase. A static vertical electric field (applied as an example in Fig. 2), as well as spontaneous polarization potential discussed throughout the text, are included in the tight-binding calculation via a potential-energy shift of the orbital energies^44,45^ The spontaneous polarization potential is calculated using a simplified approach of Ref.^17,46^.

To account for the electron-hole interaction, we solve self-consistently a pair of coupled electron-hole tight-binding equations as in Ref.^47^:

2 $$\begin{aligned} \hat{H}_{TB}{|{\psi _{el}}\rangle } - e\hat{V}_{ho}{|{\psi _{el}}\rangle }&=\epsilon _{el}{|{\psi _{el}}\rangle }\\ \hat{H}_{TB}{|{\psi _{ho}}\rangle } + e\hat{V}_{el}{|{\psi _{ho}}\rangle }&=\epsilon _{ho}{|{\psi _{ho}}\rangle }\nonumber \end{aligned}$$

where $\hat{H}_{TB}$ is the one-particle tight-binding Hamiltonian, $\hat{V}_{ho}$ ($\hat{V}_{el}$) is Coulomb potential from the hole (electron) in its ground state. Thus, the Schrödinger equation for the electron (“electron iteration”) is solved in the presence of a Coulomb potential coming from the hole, and vice-versa (”hole iteration”), where:

3 $$\begin{aligned} V_{el/ho}(\vec {R}) = \iiint \frac{e |\psi _{el/ho}(\vec {R})|^2 }{\varepsilon |\vec {R}-\vec {r}|}dV \end{aligned}$$

is solved by direct integration with:

4 $$\begin{aligned} {|{\psi }\rangle }_{el/ho} = \sum _{i=1}^N \sum _{\alpha =1}^{m} a^{el/ho}_{i\alpha } {|{i\alpha }\rangle } \end{aligned}$$

where we used notation of Eq. 1, with $a_{i\alpha }$ being expansion coefficient in atomic basis ${|{i\alpha }\rangle }$. Thus at each atomic size $\vec {R}$, assuming orthogonality of tight-binding orbitals ${|{i\alpha }\rangle }$):

5 $$\begin{aligned} |\phi _{el/ho}(\vec {R})|^2\approx \sum _{\alpha =1}^{m} |a^{el/ho}_{i\alpha }|^2 \end{aligned}$$

Next, to account for the effects of configuration mixing and exchange interaction, the excitonic spectra^48^ are calculated with the configuration-interaction (exact diagonalization) method described in detail in Ref.^20^ and in Ref.^49^. However, the electron and hole states can be either directly taken from the non-interacting (NO SCF) tight-binding calculation or from the self-consistent field (SCF) tight-binding stage of calculation, as discussed earlier in Fig. 2. We note here that preceding the configuration-interaction state with the SCF calculations should allow e.g. for a better convergence of many-body spectra and it is well established procedure that has been applied so for several various nanostructures^50,51^

The Hamiltonian for the interacting electron hole pair can be written in second quantization as follows^48^:

6 $$\begin{aligned} \hat{H}_{ex} = \sum _{i}E_i^ec_i^\dagger c_i+\sum _{i}E_i^hh_i^\dagger h_i -\sum _{ijkl}V_{ijkl}^{eh,\text {dir}} c_i^\dagger h_j^\dagger h_k c_l +\sum _{ijkl}V_{ijkl}^{eh,\text {exchg}} c_i^\dagger h_j^\dagger c_k h_l \end{aligned}$$

where $E_i^e$ and $E_i^h$ are the single particle electron and hole energies obtained at the single particle stage of calculations, respectively, and $V_{ijkl}$ are Coulomb matrix elements (Coulomb direct and exchange integrals) calculated according to the procedure given in Ref.^20^. We note that the calculation of Coulomb matrix elements presents a formidable challenge for systems with hundreds of thousands of atoms^52,53^. Here, we benefit from using the newest version of our Colombo code^49^, which performs highly efficient calculations in the Fourier space^23^. For completeness, we note that dipole matrix elements—necessary for the computation of excitonic optical spectra—are calculated using the approach given in Ref.^20,53^.

## Data Availability

The data that support the findings of this study are available within the article. Further requests can be made to the corresponding author.
